# Preparation and nutritional properties of xylooligosaccharide from agricultural and forestry byproducts: A comprehensive review

**DOI:** 10.3389/fnut.2022.977548

**Published:** 2022-09-13

**Authors:** Feng Yan, Shuangqi Tian, Ke Du, Xing'ao Xue, Peng Gao, Zhicheng Chen

**Affiliations:** College of Food Science and Technology, Henan University of Technology, Zhengzhou, China

**Keywords:** xylooligosaccharide (XOS), xylanase, nutritional properties, agricultural and forestry byproducts, application

## Abstract

Xylooligosaccharide (XOS) are functional oligosaccharides with prebiotic activities, which originate from lignocellulosic biomass and have attracted extensive attention from scholars in recent years. This paper summarizes the strategies used in the production of XOS, and introduces the raw materials, preparation methods, and purification technology of XOS. In addition, the biological characteristics and applications of XOS are also presented. The most commonly recommended XOS production strategy is the two-stage method of alkaline pre-treatment and enzymatic hydrolysis; and further purification by membrane filtration to achieve the high yield of XOS is required for prebiotic function. At the same time, new strategies and technologies such as the hydrothermal and steam explosion have been used as pre-treatment methods combined with enzymatic hydrolysis to prepare XOS. XOS have many critical physiological activities, especially in regulating blood glucose, reducing blood lipid, and improving the structure of host intestinal flora.

## Introduction

XOS are functional oligosaccharides, which are composed of 2–7 xylose molecules linked by β-1, 4-glycosidic bonds, and the relative molecular weight is generally about 200–300 kDa (1, 2). XOS have excellent physical and chemical properties, such as high heat and acid resistance (3). The sweetness of XOS is about 40%-50% of sucrose (4). The viscosity of XOS is lower than other oligosaccharides, which can reduce the water activity and improve the ability to hold water in water solution (5).

In addition to excellent physical and chemical properties, XOS are also the research hotspots of scholars from all walks of life as prebiotics (6). A large number of animal experiments have proved the beneficial effects of XOS in preventing caries, regulating blood glucose, reducing blood lipid, reducing cholesterol, preventing inflammatory, improving immunity, preventing oxidation, promoting calcium absorption, which are relating to the ability of regulating intestinal flora structure of XOS (7–16). In addition, XOS could effectively prevent obesity, cardiovascular disease, atherosclerosis, and intestinal diseases (17, 18). The International Association of probiotics and prebiotics (ISAPP) identified XOS as emergent prebiotic oligosaccharides in its latest update of its prebiotic definition (19).

Most XOS are prepared by degradation of agicultural biomass (20, 21). Typical raw materials for preparation of XOS include crop straws such as wheat and sugarcane, as well as processing byproducts such as corncob and rice husk (22). XOS can also be produced from the cotton stalk, corn straw, sugarcane bagasse, and other common agricultural wastes (23). There are three main methods of extracting XOS: autohydrolysis, acid hydrolysis, and enzymatic hydrolysis (24–26). At present, enzymatic hydrolysis preparation of XOS is the primary method (27).

XOS have great potential as food ingredients due to their price competitiveness, thermal stability and pH stability, sensory properties and multidimensional effects on human health and livestock compared with other prebiotics (28–30). Globally, XOS are mainly used in the feed industry (49.6%), followed by health and medical products (25.4%), food and beverage (23.2%), and other applications (1.8%) (31). In addition, the industry's interest in XOS is reflected in an increasing number of XOS patent applications (20). The global prebiotic ingredient market is estimated to be 4.07 billion in 2017, expected to reach $7.37 billion by 2023. The compound annual growth rate (CAGR) is 10.4% (32), and the Asia Pacific region, including China, India, and Japan, are expected to have the highest increase, exceeding 9.5% (33).

This article summarizes the research progress of preparation and purification methods of XOS in recent years and introduces the physiological activities and applications of XOS to provide the basis for the further development and application of XOS.

## Preparation of XOS

### Raw materials for XOS preparation

**Figure 1:**
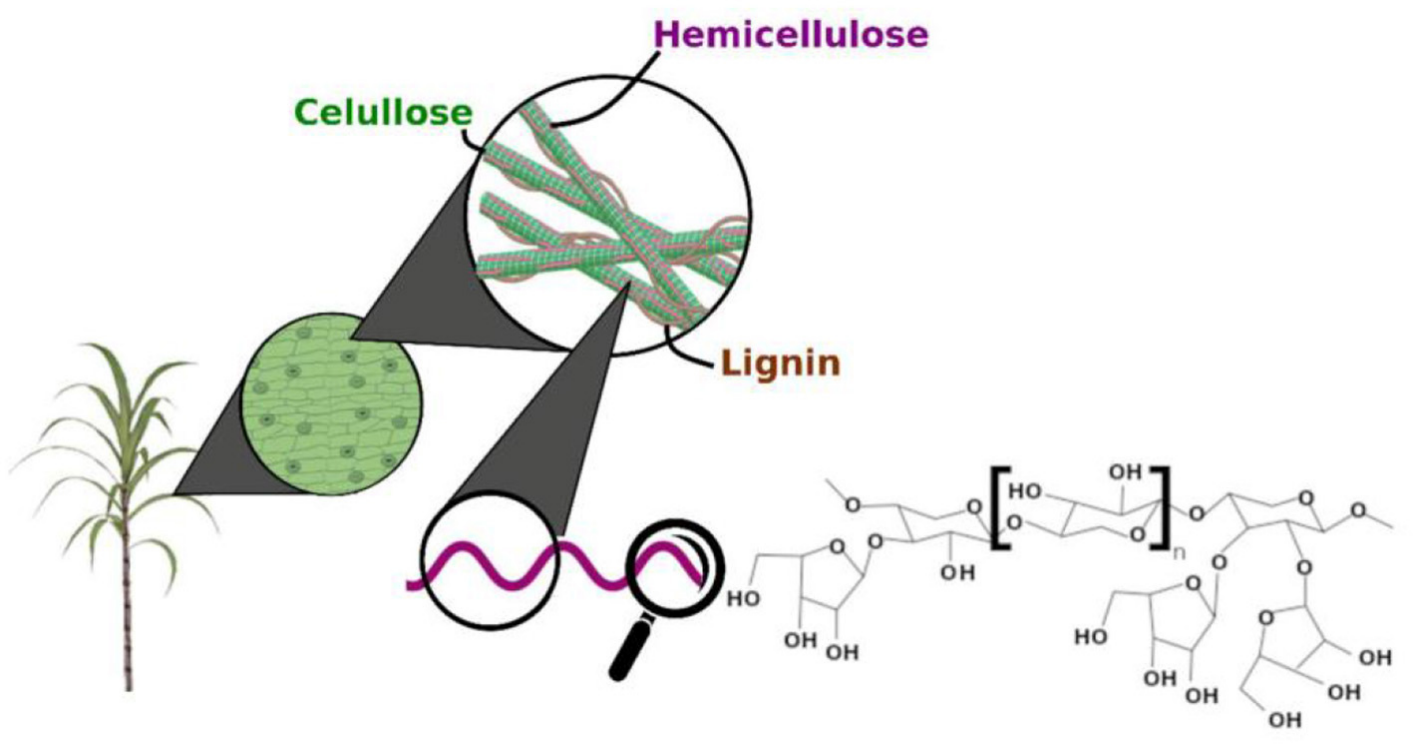
Schematic representation of the lignocellulosic biomass composition [Adopted from Capetti et al. (34)].

Figure 1 showed the schematic representation of the lignocellulosic biomass composition. Lignocellulose biomass are the non-starch part of renewable and abundant plant materials. Lignocellulose materials are mainly cellulose, hemicellulose, and lignin (35). The composition of lignocellulose varies, with an average of cellulose (30–50%), hemicellulose (20–40%), and lignin (15–25%) in the total dry matter (36). Cellulose is composed of a glucose molecular chain, which forms hydrogen bonds between different layers of polysaccharides and forms crystalline conformation. Xylan, the main component of hemicellulose, is the critical target of XOS production.

**Figure 2:**
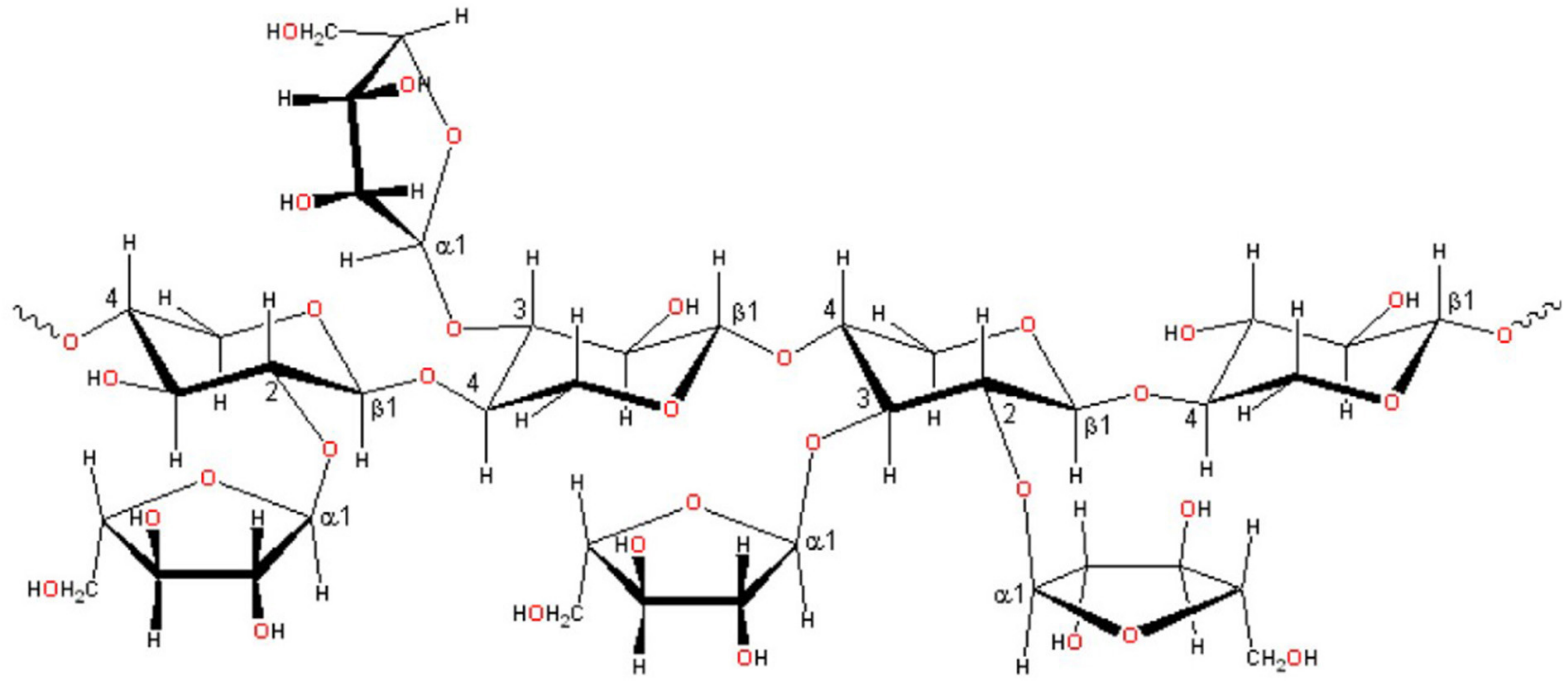
Xylan structure shows different intermolecular bonds [Adopted from Otieno et al. (7)].

Figure 2 showed the structure of xylan. Xylan is the main component of hemicellulose (60–90%), a heteropolysaccharide with a degree of polymerization (DP) between 50 and 200, containing acetyl, 4-o-methyl-dglucouronosyl, and α- substituents of arabinofuranyl residues, related to the main chain of β-1,4-linked xylopyranose units (23, 37). Table 1 lists the composition of several common lignocellulose raw materials. The higher the xylan content of the raw materials, the lower the cost of XOS production. Among these lignocellulose biomass, the hemicelluose content of corncob, sugarcane bagasse, and wheat straw are relatively high, which are ideal raw materials for XOS industrial production.

**Table 1:** Cellulose, hemicellulose, lignin composition in raw lignocellulose biomass.

**Biomass**	**Cellulose (%)**	**Hemicellulose (%)**	**Ligin (%)**	**References**
Almond shell	34.3	20.2	28.8	(38)
Big blue stem	37	28	18	(36)
Birch wood	40	24	24	(39)
Beech wood	42.5	34.3	22.2	(40)
Corncob	30–42	31–38	18–22	(37)
Coconut husk	34.1	32.6	26.0	(41)
Chestnut husk	20.6	10.5	48.3	(42)
Corn stover	40	25	17	(36)
Hazelnut shell	18.7	28.9	46.7	(43)
Miscanthus	43	24	19	(36)
Olive pomace	13.8	18.9	31.2	(44)
Olive stones	15.3	20.3	42.1	(44)
Pineapple peel	20.9	31.9	10.4	(45)
Peanut shell	20.9	19.3	42.7	(46)

### Preparation of XOS

#### Acid hydrolysis

Xylan can be hydrolyzed into soluble XOS under acidic conditions. Generally, dilute hydrochloric acid and sulfuric acid are used to hydrolyze xylan with a high degree of polymerization to produce XOS. The purpose of acid treatment is to improve the hydrolysis degree of hemicellulose, to improve the yield of XOS. Hemicellulose is separated into oligosaccharides and monosaccharides with a wide range of DP through the breaking of glycosidic bonds of xylose (37, 47). However, acid hydrolysis leads to equipment corrosion, which limits its use. In addition, acid hydrolysis will produce excess xylose and other toxic reaction products at high temperatures, such as furfural and hydroxymethylfurfural (HMF), which are harmful to food applications (48, 49).

It was reported that the yield of XOS obtained by hydrolyzing poplar wood with 5% acetic acid at 170°C was 39.8% (50). Ying et al. elucidated that the increase of sulfuric acid dosage enhanced the lignin removal of poplar pretreated with hydrogen peroxide acetic acid (51). The maximum XOS yield was 68.5% when XOS were produced by hydrolyzing corncob with 5% propionic acid at 170°C for 50 min (52). It was reported that the product obtained by hydrolyzing brewer's grains with 1.85% sulfuric acid for 19.5 min contains 6.6 g/L *arabinoxylooligosaccharide* (AXOS) (53). The acetic acid pre-treatment of poplar could effectively produce XOS, with a yield of 55.8%, and acetylation degradation of lignin occurred after acetic acid pre-treatment (54).

Acid hydrolysis to obtain XOS has been widely used because it was a fast and easy technology (21). High XOS yield could be obtained by hydrolysis with sulfuric acid of lignocellulose biomass. However, the yield of oligomers is lower than monomers, mainly due to the higher yield of xylose (6, 55). Even some methods change the acid conditions, improve the yield of XOS and optimize the existing preparation process; but the acid hydrolysis efficiency is still not high; there are still many impurities in the prepared products; and the content of XOS is still low.

#### Autohydrolysis

Agricultural plant biomass rich in xylan can also be directly hydrolyzed under high temperature and high pressure to produce XOS. Autohydrolysis is a non-chemical process, which refers to the deacetylation of D-xylan at high temperature in the presence of water (56). Autohydrolysis occurs under slightly acidic conditions due to the partial cleavage of acetyl groups in plant cell walls to form acetic acid (57). In the process of autohydrolysis, XOS are typical reaction intermediate, and their concentration depends on the balance between the decomposition of polymer hemicellulose into XOS and their further decomposition into monomer xylose. Therefore, under medium conditions, the yield of XOS will be higher. Treatment with increased severity resulted in decreased DP and increased decomposition of XOS into xylose. Hemicellulose is easily affected by water under high pressure and high temperature. Exposure of lignocellulosic biomass to water causes hemicellulose to penetrate the cellular structure, resulting in cellulose hydration and hemicellulose depolymerization. The action mode of hydrothermal treatment of lignocellulosic biomass was in the subcritical region of water (100–374°C) (58).

Autohydrolysis is heat treatment with steam or liquid water at high temperature or high pressure (55, 56). Under the autohydrolysis, the autoionization of water will produce ions, which leads to the depolymerization effect of hemicellulose (59). Acetic acid is usually added during autohydrolysis to increase the formation of hydrogen ions (23). The yield of XOS is the high under moderately severe operating conditions (60). It was reported that the maximum yield of XOS (55.3 wt%) was obtained by hydrothermal treatment of pecan shells at 160°C for 2 h. At the same time, high temperature (220°C) and short time (0.5 h) were helpful in hydrolyzing XOS with high DP, in which the yield of XOS (DP2-6) was 37.5 wt% (61). The autohydrolysis of almond shells (200°C, 5 min) resulted in low DP, and the concentration of XOS (xylobiose and xylotriose) was only 3.5% (38). Small-scale (150 tons of brewery waste grain per day) biological refineries could make profits by valuing the waste grain produced by large breweries and applying high-solid hydrothermal technology to produce high-value products xylitol and XOS (62). It was also elucidated that the recovery rate of high-purity polymeric hemicellulose with molecular weight (21–30 kDa) was 35–37% when high-purity hemicellulose (xylan) was partially extracted from wood waste by alkali mediated hydrothermal method; the separated hemicellulose could be chemically transformed into high-value commercial products, such as prebiotics (XOS) (63).

The main advantage of autohydrolysis method is that it has low or no requirements for corrosive compounds and is marked as an environmentally friendly process (36). In the past decades, hydrothermal treatment has been widely studied as the first step of biorefinery because of its environmentally friendly advantages and the selectivity of dissolving hemicellulose as oligosaccharides over other treatments (64, 65). Hydrothermal pre-treatment is considered an ecologically friendly and inexpensive alternative method to treat lignocellulose (66, 67). Autohydrolysis technology automatically ionizes water into hydrogen ions, allowing hemicellulose compounds to be released from lignocellulose, such as acetyl groups in acetic acid. This organic acid acts as a mild catalyst during the reaction, which is conducive to the subsequent dissolution of other hemicellulose-derived compounds (68, 69). Therefore, hydrothermal treatment is a technology to reduce the corrosion effect and cost of different solvents, and has high selectivity for hemicellulose.

Although the consumption of chemicals is low, due to the high pressure and temperature conditions, autohydrolysis process requires high energy consumption. The green characteristics of autohydrolysis will also depend on the energy used (70). It was reported that the most common temperature range to achieve high yield and minimum degradation of compounds was about 160–180°C (42, 71). The high temperature usually causes the release of many monomers (xylose) and impurities, such as furfural and HMF produced by sugar degradation, as well as the phenolic compounds produced by lignin (72, 73). The acidic hydrogen ion is formed due to the release of acetyl group in lignocellulosic biomass; acetyl group is the catalyst for hemicellulose depolymerization (71). The depolymerization of oligomers begins with the random breaking of the bond of xylose, producing oligomers short enough to be extracted from the biomass structure (74).

The main disadvantage of heat treatment is still to produce a large number of unwanted byproducts, such as other oligomers, monosaccharides, acetic acid, furfural, HMF, formic acid, levulinic acid, phenolic compounds, etc., (30, 75). It was observed that the degradation compounds released from the mixed biomass of hydrothermal treatment, had a significant inhibitory effect on the growth of *Lactobacillus brevis*. The dissolved lignin concentration of 1 g/L inhibited the growth of *Lactobacillus brevis*. After the adsorption purification step using Amberlite XAD 16N resin, the purified XOS showed the exact cell yield and product yield as commercial XOS (76). In general, a separation process is required to remove unwanted compounds. The existence of the former compound in XOS mixture leads to serious purification difficulties, and then increases the production cost (56).

The release of degraded compounds depends on the composition of biomass and process conditions. Generally speaking, a separation process is required to remove unwanted compounds. The presence of the former compound in the XOS mixture leads to serious purification difficulties and consequent increased production costs (56). Another disadvantage of using autohydrolysis to produce XOS is that special equipment is required due to high temperature and high pressure (23, 24).

#### Enzymatic hydrolysis

**Figure 3:**
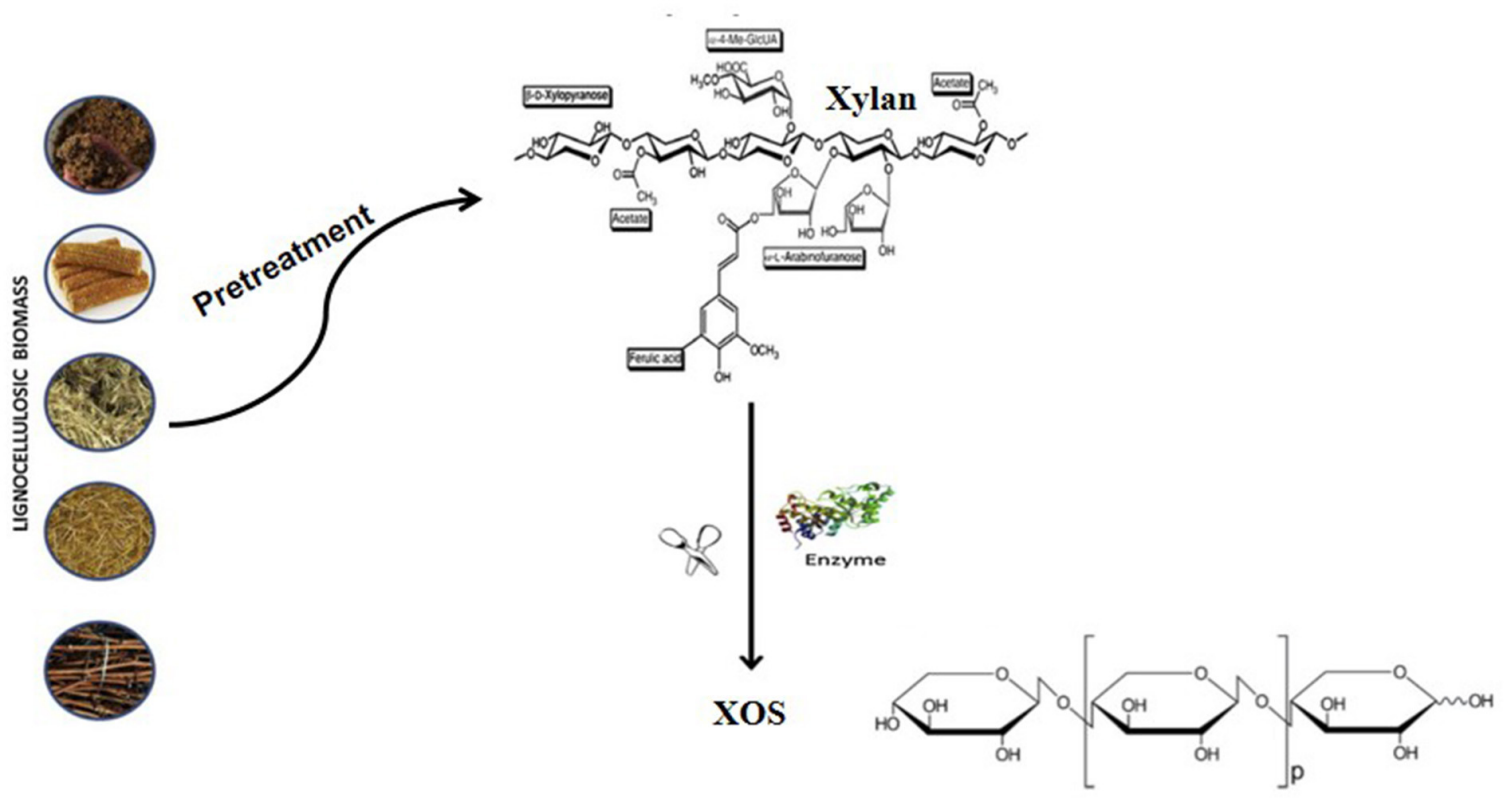
Schematic representation of XOS manufacture by enzymatic method.

Figure 3 showed the xylanase hydrolysis of lignocellulose. XOS can also be produced by enzymatic hydrolysis of xylan. Compared with acid hydrolysis and autohydrolysis, enzymatic degradation is an ideal XOS production method because it has specificity and the least byproducts. In addition, enzymatic hydrolysis does not require any special equipment. The current commercial process uses an enzyme process, which has mild operating conditions, and does not use toxic chemicals, so enzymatic hydrolysis is more in line with the viewpoint of biodegradation process. In addition, the use of xylanase is efficient and specific, allowing higher control of DP and reducing the production of unwanted xylose and other byproducts (77).

Enzymatic hydrolysis is usually used for the extraction of XOS due to the mild enzymatic hydrolysis conditions and high product quality (78). However, the isomerization of lignocellulosic materials will be seriously affected by the structure of composite lignin-hemicellulose. Therefore, it is essential to destroy the composite structure to expose more hemicellulose to improve the extraction efficiency. Physical and chemical pre-treatment technology have been used before enzymatic hydrolysis. In addition, the source of materials and xylanase have an impact on enzymatic hydrolysis.

##### Physical pre-treatment

Physical pre-treatment mainly includes the hydrothermal method, steam explosion method, ultrasonic method, and microwave method.

Steam explosion is instantaneous blasting under high temperature and high pressure. The hemicellulose recovery of corn cob after steam explosion at 196°C for 5 min was 22.8% (79). After a steam explosion at 204°C for 4 min and 180°C for 30 min, the yield of XOS in wheat straw was 8.9 and 13.9/100 g (72, 80). Steam explosion was also applied to the pre-treatment of rice husk, and the final output of XOS was 17.35 mg/mL xylan (81). In addition, after the steam explosion, 40% of xylan was degraded into XOS, and the degree of polymerization of steam explosion hydrolysates had good prebiotic properties (82).

The hydrothermal method processes materials in high-temperature or high-pressure hot water. It was reported that the xylan yield of wheat straw reached 56.2 g/kg after hydrothermal pre-treatment at 180°C for 40 min (39). After hydrothermal pre-treatment at 190°C, 1.8 MPa for 13 min, the extraction rate of xylan from corncob was 18% (6). The extraction rate of xylan was 23.82/100 g from dry corn straw subjected to non-isothermal hydrothermal pre-treatment (83). The extraction of XOS from corn fiber by hydrothermal pre-treatment at 160°C was also reported (84).

Ultrasonic and microwave had been applied to the pre-treatment of lignocellulose materials. Under 121°C ultrasonic pre-treatment, 39% of xylan in corncob was released, which was higher than the conventional extraction method (34%), and the extraction time shortened from 24 h to 43 min (85). After microwave pre-treatment at 185°C for 10 min, the recovery rate of xylan was higher than that of high-pressure steam pre-treatment, because microwave pre-treatment was easy to control the degree of reaction (86).

The results showed that physical pre-treatment was helpful to the release of xylan and XOS. The steam explosion can significantly improve the release of XOS, which has the advantages of simple operation, no pollution, low energy consumption, and a short production cycle. Therefore, the steam explosion is a promising pre-treatment method for XOS extraction in the future.

##### Chemical pre-treatment

Chemical pre-treatment mainly uses acid and alkali to extract xylan from lignocellulosic materials, and the yield of xylan varies according to the source of materials and extraction conditions. Table 2 lists the material sources, extraction conditions, and pre-treatment methods.

**Table 2:** Material sources, conditions, and xylan yield of commonly used chemical pre-treatment methods.

**Material sources**	**Extraction method**	**Xylan yield**	**References**
Corn cob	12% NaOH	83% of original xylan	(6)
	Acidic electrolyte water, pH 2.0	55%	(87)
	4% NaOH and methyl alcohol	11%	(88)
	10% NaOH, 75°C, 90 min	20%	(89)
Wheat straw	0.5 mol/L NaOH, 55°C, 2 h	49.3% of original xylan	(90)
Corn stalks	10% NaOH+1% NaHBH_4_, 20°C, 10 h	54% of original hemicellulose	(91)
Corn husks	12% NaOH, 121°C, 0.2 MPa, steam 45 min	84.60 ± 2.19% of original xylan	(92)

As shown in Table 2, the most commonly used chemical pre-treatment method was alkali extraction, which mainly used 5–24% sodium hydroxide and potassium hydroxide as extraction solvents. However, the yield of xylan was relatively low when alkali extraction is used alone. Auxiliary methods such as steam and ultrasound are needed to improve the yield of xylan. Xylan was extracted from corncob with sodium hydroxide and methanol solvent, and the extraction rate reached 11% (89). A similar xylan yield can be obtained when xylan is extracted from dry corncob with 10% sodium hydroxide at 75°C (89). The yields of XOS were 83 and 84.5%, respectively, when xylan were extracted from corncob and corn shells with 12% sodium hydroxide solution with the steam pre-treatment (6, 92). Ultrasonic assisted alkali extraction can significantly improve the yield of XOS in corncob, and the content of related XOS can reach 174.81 mg/g matrix (93).

Due to the low efficiency of acid extraction, acid extraction was rarely used and was usually assisted by physical methods. Xylan was extracted from corncob by acid electrolyte water (pH 2.0) combined with the steam explosion, and the extraction rate was 55% (87). After pretreated corncob with dilute acid and alkali to extract the lignin-saccharide complex, the yeild of XOS was 5.80 ± 0.14 mg/mL from the complex by xylanase hydrolysis (94). According to reports, after being dissolved in 1.0 M H_2_SO_4_ for 12 h, XOS were extracted from corncob by high-pressure hydrolysis, and the maximum yield was 67.7% (95).

The alkali extraction method is greatly affected by temperature and the extraction rate increases with the increase of temperature. The alkali extraction method does not produce byproducts, which is better than the acid extraction method, but it have high requirements for equipment. Therefore, it is suggested to use physical processes (such as steam and ultrasonic) and chemical processes to improve the yield of xylan.

##### Xylanase hydrolysis

Xylanase systems include endo-xylanase and xylose releasing enzyme exo-xylanase, or β-xylosidase, and debranching enzyme (30, 96). For the production of XOS, only endo-xylanase are meaningful. Based on sequence conservation, these enzymes can be found in glycoside hydrolase families (GH) 5, 8, 10, 11, and 43. In addition, exo-xylanase or β-xylosidase preparations with low activities were needed to avoid xylose production (56). Xylanases have been isolated from many different fungi and bacteria, but most commercial xylanase hydrolysates are currently produced by transgenic xylanase strains (97, 98).

It was reported that the main product was xylobiose after the cauliflower stalk was hydrolyzed by natural endo-xylanase extracted from *Aspergillus niger* TCC9687. The cauliflower stalk XOS showed significantly high antioxidant and antibacterial activities and reduced the viability of human bone cancer MG-63 cells, both alone and in combination with (*Lactiplantibacillus plantarum, Bifidobacterium bifidum, Lactobacillus delbrueckiissp*. *Helveticus*); the antibacterial components of cauliflower stalkm XOS were dihydroxybenzoic acid and aspartic acid (99). Abdella et al. reported that after the xylanase produced by *Paecilomyces wallichii* was applied to beech xylan to produce different types of XOS; when the extract concentration was from 0.1 to 1.5 mg/mL, the antioxidant activity of XOS increased from 15.22 to 70.57% (100). XOS from oil palm empty fruit bunch hemicellulose produced by xylanase from *Thermomyces cyanobacterium* hydrolysis was composed of xylotriose and xylobiose. XOS was evaluated as the substrate of two probiotics (*Lactobacillus plantarum* WU-P19 makes better use of XOS than *Bifidobacterium* TISTR2129) found in the human gastrointestinal tract (101).

Table 3 lists the hydrolysis and yield of different raw materials with endo-xylanase from different sources. It can be seen from Table 3 that the yield of fungal xylanase is high. It is reported that bacteria such as bacillus and streptomyces could also produce xylanase (114). Recombinant xylanase could also obtain a relatively high yield, but large-scale natural production of recombinant enzyme required a highly complex purification process, which significantly increases the cost. Enzymatic hydrolysis of xylan could also be achieved *in situ* by microbial fermentation. In this process, bacteria were cultured to produce xylanase and secreted into the reaction medium, where the enzyme hydrolyzes xylan to produce XOS (115).

**Table 3:** Xylanase used for XOS production, operational conditions, and yield.

**Xylanase**	**Biomass substrate**	**Xylanase ratio**	**Operational conditions**	**Hydrolysis yield**	**References**
Wild-Type endo-xylanase	Paper mulberry pulp	125 U/g	53.8°C, 12 h	1.23 ± 0.09 g/L	(102)
Recombinant endo-xylanase	Paper mulberry pulp	125 U/g	53.8°C, 12 h	1.59 ± 0.07 g/L	(102)
Cellulase-Free xylanolytic enzyme from *Bacillus firmus* K-1	Corn cob	3 U/mL	50°C, pH4.8, 4 h	44.6% (intial xylan)	(103)
Endo-Xylanase from *Aspergillus niger* MTCC 9687	Lauliflower stalk	20 U/g	50°C, 5h, pH5.4	7.4 mg/mL	(104)
Crude fungal xylanases from A. flavus KUB2	Spent mushroom	20 U/g	50°C, 5h, pH5.4	1.37–1.48 mg/mL	(105)
GH10 from *Caldicellulosiruptor bescii* xylanase	Rice straw xylan Sugarcane bagasse	300 U/mL	50°C, pH 6.0, 72 h	2.93 mg/mL 1.12 mg/mL	(106)
GH11 from *Bacillus firmus* K-1 xylanase	Rice straw xylan Sugarcane bagasse	300 U/mL	50°C, pH6.0, 72 h	1.79 mg/mL 1.10 mg/mL	(106)
Two recombinant endo-xylanase from *Streptomyces thermos*-Riseus (StXyl10, StXyl11)	Red alga dulse	0.5 μg/mL Then 2.0 μg/mL	StXyl10 (50°C, 4 h) StXyl11(60°C, 36 h)	95.8% (intial xylan)	(107)
Crude xylanase produced with *Aureobasidium pullulans* NRRL Y−2311–1 from wheat bran	Autohydrolysis of hazelnut shells	240 U/g	50°C, pH 6.0, 24 h	22.5 g/L	(108)
Combinations of endo-β-(1,4)-D-xylanase enzyme with accessory enzymes (α-L-arabinofuranosidase, feruloy-esterase, and acetylxylan-esterase)	Barley straw	Endo-β-(1,4)-D-xylanase NS50030 7.2 U/mL; α-L-arabinofuranosidase 6.3 U/mL; feruloylesterase 0.05 U/mL, and acetylxylan esterase 5 U/mL	50°C, pH 4.8, 5 h	13.6 g XOS/100 g	(109)
Commercial xylanase	Rice husk arabinoxylan	50 U/g	50°C, pH 5.5 24 h	64.01%	(110)
Commercial xylanase	Rice straw arabinoxylan	100 U/g	50°C, pH 5.5, 24 h	59.52%	(110)
Crude xylanase from *Aureobasidium pullulans* CCT 1261	Beechwood xylan	260 U/g	40°C, pH 6.0 24 h	10.1 mg/mL	(111)
*Aspergillus versicolor* endo-xylanase	Xylan from sugarcane bagasse 0.17% substrate	65 UI/g	55°C, 24 h	67.43%	(112)
*Aspergillus versicolorendoxylanase*	Xylan from sugarcane leaf 0.17% substrate	65 UI/g	55°C, 24 h	69.71%	(112)
Xylanase complex fermentation by *Aspergillus niger*	Sugarcane extracted xylan	5 U/mL	55°C, pH 5.8, 1 h	3.1 g/L	(113)

Xylanase hydrolysis process is relatively soft and easy to purify. There is no apparent other production, and the color of XOS is relatively light. XOS prepared by enzymatic hydrolysis have good prebiotic potential and antioxidant performance. In addition, the use of xylanase has high efficiency and specificity, allowing higher control of DP and reducing the production of unwanted xylose and other byproducts. At present, xylanase hydrolysis is the primary method to produce XOS.

To sum up, the existing methods have optimized the preparation of XOS to a certain extent and improved product efficacy. In recent years, the industrial application of XOS has been greatly limited due to high content of impuritie XOS; and the product quality of XOS was not easy to control. Therefore, the refining, separation, and purification of XOS have also become the key to subsequent industrial application. At present, the development of XOS has not reached its peak. As a new generation of functional sugars, XOS have not been fully used. These production optimizations have promoted the application and development of XOS and laid the primary theoretical foundation for large-scale popularization and use in the future.

### XOS purification

After XOS production, undesirable compounds and oligosaccharides were produced (116). The presence of unwanted compounds such as glucose and xylose will increase the calorific value of XOS and change their sweetness ability (56). On the other hand, the prebiotic effect of XOS also seems to depend on their purity level. It has been observed that high-purity XOS products have a more significant impact on biological function (57).

To remove unwanted components and obtain high-purity XOS, subsequent purification treatment is required (28). In particular, more components will effect the purity of product when the autohydrolysis method is adopted to treat lignocellulose (117).

The commonly used purification methods include adsorption separation, solvent extraction, membrane separation, and chromatographic separation.

Adsorption on the active solid surface is usually used in combination with the solvent elution step to separate oligomers from monomers and remove other unwanted pollutants. Commonly used adsorbents for purifying XOS include activated carbon, acid clay, bentonite, diatomite, aluminum hydroxide oxide, titanium, silica, and porous synthetic materials (1, 56, 118). Among them, activated carbon is the most commonly used evaluation method, whether in solution or fixed bed adsorption. Activated carbon treatment has proven to be a viable option for removing extract-derived, lignin-derived, and carbohydrate degrading compounds present in XOS mixtures (119). On the other hand, ion exchange resins are combined with different purification strategies to remove salts, heavy metal ions, charged organic compounds and pigments in XOS mixtures (56, 120).

Solvent extraction mainly removes the non-sugar components from the hydrolysate. The recovery and purification degree of the XOS mixture depend on the solvent used for extraction. Ethanol, acetone, and isopropanol are the most common options for refining crude XOS solutions (121–123). In XOS production, solvent extraction is usually used to recover hemicellulose derivatives from pre-treatment (55). In this case, vacuum evaporation is generally used in the first stage to remove volatile compounds and concentrate XOS solution (56). On the other hand, organic solvent precipitation allows the recovery of XOS or xylose while removing phenols and extracting derived compounds.

Chromatographic separation for XOS purification produces analytical grade high-purity components. Gel permeation chromatography (GPC) (124), water-soluble exclusion chromatography (SEC) (125), ion-exchange chromatography (IEC), and centrifugal partition chromatography (CPC) are some standard technologies for purifying XOS (126, 127), Ho et al. used GFC to purify XOS produced by autohydrolysis of agricultural residues. In this cases, GFC could effectively remove high DP oligosaccharides. More importantly, GFC could remove unwanted small molecules, such as monosaccharides, acetic acid, and degradation compounds (furfural, HMF, and phenol) (128).

Membrane separation is another powerful technique commonly used for oligomer purification. Ultrafiltration and nanofiltration based technology is the most promising processing strategy for manufacturing high-purity and concentrated oligosaccharides (55). The popularity of this technology can be attributed to its low energy consumption requirements, relatively easy amplification, and easy operation variables (13, 116, 129–131). Membrane technology is currently considered to be the most promising strategy for industrial production of high-purity XOS. In this case, the ultrafiltration separation of oligosaccharides from high molecular weight compounds has low energy consumption and is easy to operate and enlarge (128). However, its disadvantage is that its performance is poor when small molecules must be removed.

Meanwhile, purification strategies with different properties are often used in combination to improve the purification of XOS. It was reported that a combination of nanofiltration, solvent extraction and dual ion exchange chromatography method could achieve 90.7% XOS purity (132).

## Nutritional properties of XOS

**Figure 4:**
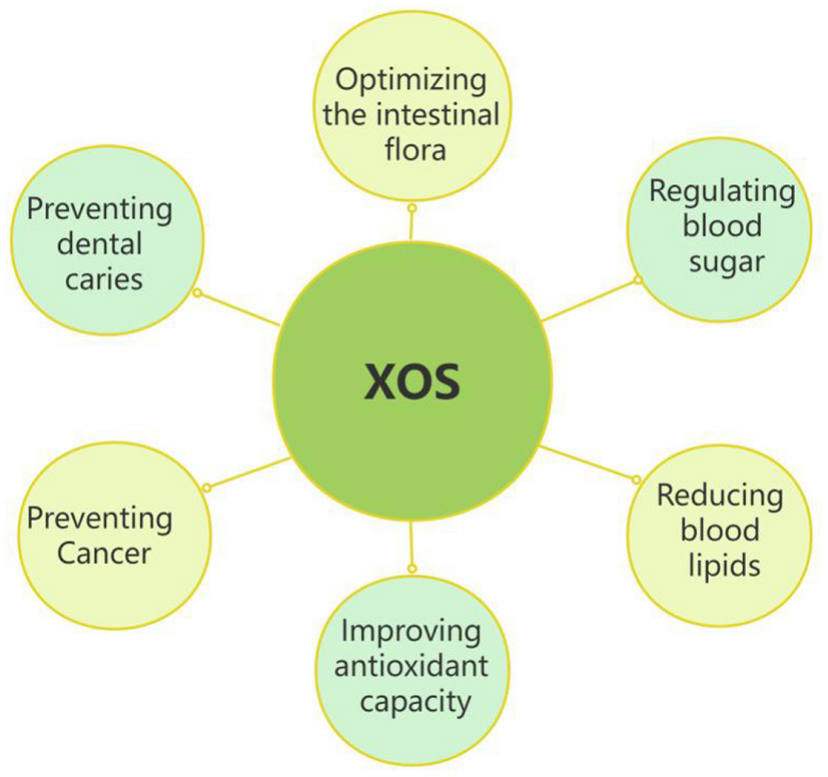
Nutritional properties of XOS.

Figure 4 showed the main nutritional properties of XOS. XOS have many important physiological activities, especially in regulating blood glucose, reducing blood lipids, improving antioxidant capacity, preventing cancer, preventing dental, and improving the structure of host intestinal flora.

### Optimizing the intestinal flora

**Figure 5:**
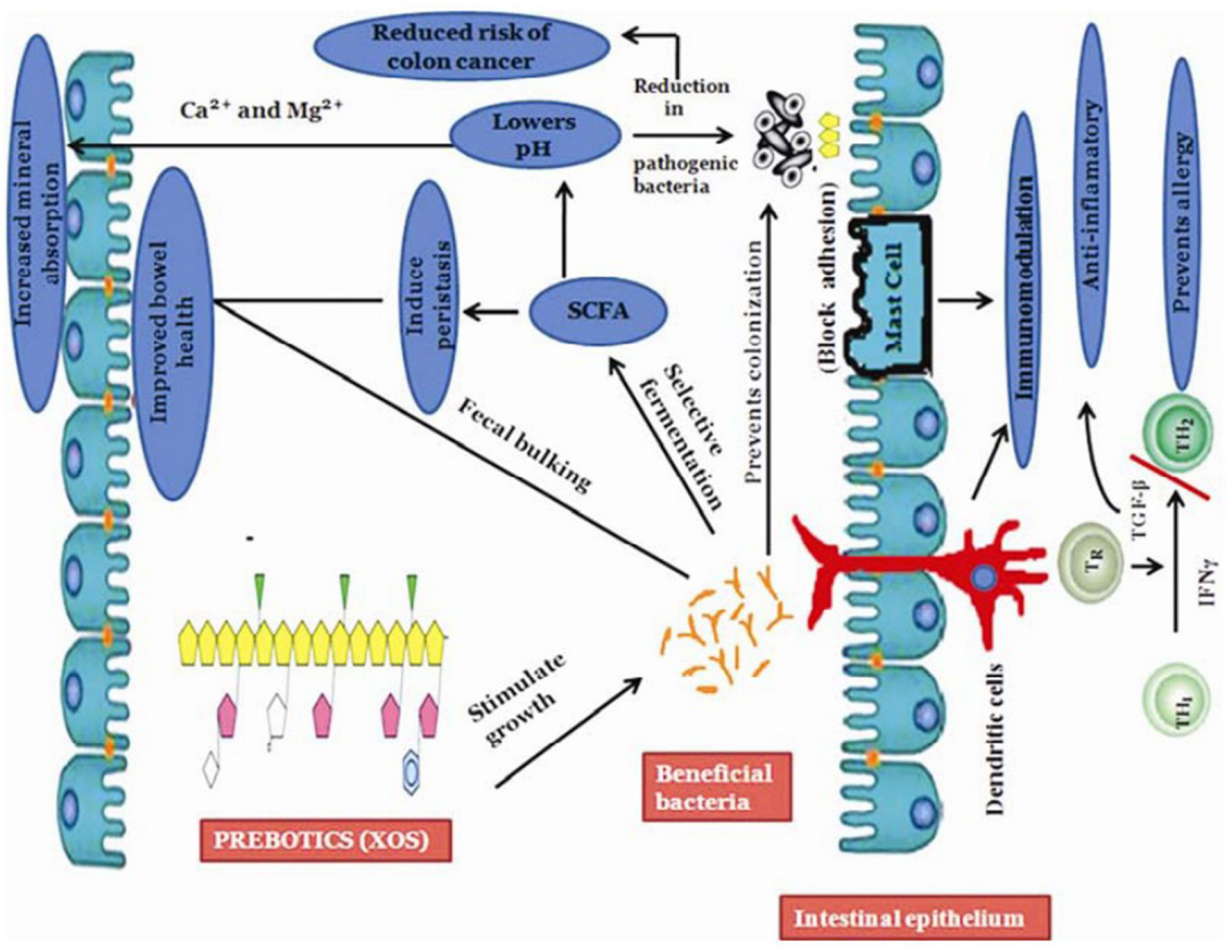
Illustration of benefits incurred by prebiotics on immune system: Stimulates growth of the beneficial bacterium in the large intestine that prevents colonization of harmful bacteria; increases production of short-chain fatty acids (SCFA) and helps in improving bowel health that reduces risks of colon cancer [Adopt from Slizewska et al. (133)].

Figure 5 showed the role of XOS in regulating intestinal flora. XOS can change the composition of intestinal microorganisms, increase the number of probiotics and produce healthy fatty acids. It was found that XOS from giant awn could increase the number of *Bifidobacteria, Lactobacillus* and *Escherichia coli* without affecting the number of pathogenic bacteria such as *Clostridium perfringens* (134). XOS extracted from corn straw also had a significant effect on the proliferation of *Lactobacillus* and *Bacteroides* (80). The addition of XOS during fattening period would increase the concentration of acetic acid, linear fatty acids and short-chain fatty acids in pig intestinal contents, and change the composition and metabolic activity of intestinal flora (135). The intake of XOS could significantly increase the number of *Bifidobacteria* in human intestine (136). It was elucidated that *in vitro* fermentation of XOS from birch could significantly proliferated the number of *Bifidobacteria, Staphylococcus*, especially *Staphylococcus hominis*, which could produce *bacteriostasis* and inhibit corresponding pathogenic bacteria such as *Staphylococcus aureus* and *Helicobacter pylori* (137). Hald et al. elucidated that after ingestion of arabinoxylan, the number of *Bifidobacteria* in feces increased significantly, while the number of *Lactobacillus, Clostridium*, and *Akmann mucophilus* did not change significantly (138).

The reason that XOS selectively proliferate beneficial bacteria such as *Bifidobacteria* in the intestine is related to the production of vitamins and immune stimulation (139). The proliferation of *Bifidobacteria* in the intestine will also inhibit the growth and reproduction of pathogenic bacteria, produce some digestive enzymes and help the body rebuild the intestinal flora (140). The effect of XOS on intestinal health is also reflected in the large production of organic acids, such as short-chain fatty acids, acetic acid, propionic acid, and butyric acid, as well as other organic acids such as lactic acid, succinic acid, formic acid, isobutyric acid, valeric acid, caproic acid, and isohexanoic acid (99, 141). These organic acids play essential roles in preventing various intestinal diseases. The increase in acetic acid concentration was particularly significant after ingestion of Arabinoxylooligosaccharide (136). It was reported that the proliferation of *Bifidobacteria* caused by the intake of XOS is an essential reason for maintaining intestinal health and preventing intestinal diseases (30). The study found that after the intake of XOS, the number of *Enterobacteriaceae* and *Clostridium perfringens* decreased significantly, which effectively reduced the incidence of intestinal diseases caused by harmful bacteria (88, 142).

XOS can selectively proliferate beneficial bacteria for three reasons: (1) Providing energy materials for beneficial bacteria (143). (2) Proliferating beneficial bacteria form a microbial barrier to prevent pathogen colonization (144). (3) XOS are fermented and utilized by *Bifidobacteria* and other microorganisms in the intestine, and the organic acids produced reduce the pH of the intestine, and most pathogenic bacteria are suitable to grow in a neutral environment to inhibit the growth and reproduction of pathogenic microorganisms (145).

### Regulating blood sugar

XOS have unique molecular structure of β-1, 4 glycosidic bonds so that the enzymes in the digestive tract in the body cannot decompose them and cannot be directly absorbed and utilized by the human body, so they do not affect the blood glucose concentration. XOS cannot be digested and absorbed by the animal gastrointestinal tract but can be fermented and utilized by beneficial bacteria such as *Bifidobacteria* in the intestine in the large intestine and produce a large number of organic acids such as short-chain fatty acids (146). It was found that type 2 diabetic patients had significantly lower blood sugar levels after 8 weeks of XOS intake (147). The intake of 5% XOS could significantly reduced the blood glucose of obese mice (146). XOS from cereals could effectively improve the blood glucose level of mammals (148).

It was reported that XOS regulated blood glucose and lipid metabolism in mammals depending on their fermentation process in the colon (148). Some researchers argued that XOS could improve glucose tolerance by reducing plasma glucose levels and enhancing insulin sensitivity (149). It was reported that after XOS were ingested by mammals, a large amount of propionic acid produced by the fermentation of beneficial bacteria in the intestine could stimulate the production of glucagon like peptide 1 (GLP-1), which stimulates the secretion of insulin, thereby increasing the synthesis of liver glycogen and reducing the level of plasma glucose (150).

To sum up, XOS play a regulatory role in blood glucose levels. For people with high glucose, diabetes, or impaired glucose tolerance, XOS intake has a positive effect on lowering blood sugar levels, which is consistent with most studies.

### Reducing blood lipids

Many studies have shown that XOS could effectively reduce the lipid levels of obese people. For example, it was found that after 8 weeks of continuous intake of 4 g/d xylose, fat in patients with type 2 diabetes decreased significantly (147). XOS could reduce the levels of total cholesterol, low-density lipoprotein, triglyceride, and increase the level of high-density lipoprotein in obese mice with a high-fat diets (146). XOS could also reduce the fat level of broilers (151).

The intake of oligosaccharides will reduce the levels of total auxin and acylated auxin. In contrast, the reduction of acylated auxin will reduce food intake to improve obesity and control metabolism. It was found that the short-chain fatty acids produced by microbial fermentation of XOS in the intestine affect the metabolism of cholesterol, in which propionic acid was absorbed by the intestine and entered the blood through the portal vein to the liver to reduce the synthesis of cholesterol in the liver and improve the sensitivity of insulin to regulate the body's lipid metabolism (152). In addition, some studies have explained the mechanism of XOS reducing blood lipid from the perspective of bile acid. It was found that the mechanism that XOS reduced the cholesterol level of patients with high cholesterol was the excessive excretion of bile acids (153).

### Improving antioxidant capacity

XOS also have antioxidant activity (154). The antioxidant activities of XOS are mainly reflected in increasing the content of non-enzymatic antioxidant substances and improving the activity and level of antioxidant enzymes (147). It was found that XOS could significantly reduce the levels of oxidized glutathione (GSH) and malondialdehyde (MDA) in serum, heart and liver of high-fat diet mice, and normal mice (146). XOS intake could significantly increase the activity and level of antioxidant enzymes such as SOD (superoxide dismutase), CAT (catalase), and GSH PX (glutathione peroxidase) in the heart of mice with a high-fat diet (155). Abasubong et al. reported that 5% XOS (the mass fraction of 1.5%) could significantly improve the growth performance, antioxidant capacity, innate immunity, and hydrophilic bacilli resistance of *Sparus macrocephalus* (156). XOS could increase the contents of *Lactobacillus* and *Bifidobacterium* in mouse feces and reduce the contents of *Enterococcus, Enterobacter*, and *Clostridium*. The vitro antioxidant results showed that the conbination of XOS and *Lactobacillus plantarum* had free radical scavenging activity (154).

### Preventing cancer

Short-chain fatty acids and other organic acids produced by XOS fermentation in the intestine have a specific role in preventing cancer, and their immune regulation in the body are essential means to prevent cancer (146, 157). Studies have shown that XOS could change the intestinal microbiota of mice and improve the intestinal barrier (158). It was demonstrated that XOS could reduce systemic inflammation, increase trabecular thickness, reduce osteoclasts and active erosive surfaces, and restore the rate of mineral deposition and bone formation in male *Wistar* rats (159). Yin et al. found that the inflammatory state and intestinal barrier of XOS-fed piglets improved significantly (160). Many reports have reported that the addition of oligosaccharides could reduce the expression of TNF-α (tumor necrosis factor) and NF-κβ (proinflammatory nuclear transcription factor protein) in the colon (161). It was also verified the anti-inflammatory activity of wheat arabinose oligosaccharides (162). XOS could also significantly increase the activation potential of T cells and B cells in tumor-bearing mice, as well as the immune ability in body fluids and cellular mediators, and play an anti-tumor role. It was also reported that the taking of XOS could minimize the risk of colon cancer, produce cytotoxic effects on leukemia cells, improve the immune system, and has a positive effect on type 2 diabetes (40, 163–165).

Beneficial bacteria can regulate immune factors and antibodies by using organic acids produced in the process of XOS to improve the immune function of the body (166). *Bifidobacterium* can increase the number of peripheral leukocytes so that the immune function is enhanced through the proliferation of *Bifidobacteria* (154). At the same time, XOS can increase the number of blood monocytes, serum alkaline phosphatase activity, and lysozyme activity. As an immune adjuvant, *Bifidobacterium* can recognize PP lymph nodes, activate intestinal lymph nodes, and induce lymphocyte outflow through lymphatic vessels; the immune system of the body is activated through lymphatic circulation (167). In addition, oligosaccharides directly bind to sugar receptors on the surface of immune cells to stimulate immune cell differentiation and increase activity; XOS can also be used as foreign antigens to effectively and permanently stimulate the immune system and promote the cell division and development of immune organs (153).

### Other nutritional properties of XOS

At the same time, XOS can also prevent dental caries (168). XOS can not be decomposed by *Streptococcus mutans* and other bacteria in the mouth. The absorption rate of calcium was improved when XOS and calcium were ingested simultaneously (161). Kobayashi et al. found that the use of acidic XOS in mice with iron deficiency anemia significantly reduced the contents of ferritin in the liver and iron transporter in the small intestine, indicating that XOS could improve the body's iron absorption capacity and promote the body's absorption of minerals. XOS could promote the proliferation of beneficial bacteria in the intestine; and the enzymes (such as phytase) produced by the beneficial bacteria promoted the dissociation of mineral ions in the intestine and improved the intestinal absorption rate of the mineral (169).

## Application of XOS

**Figure 6:**
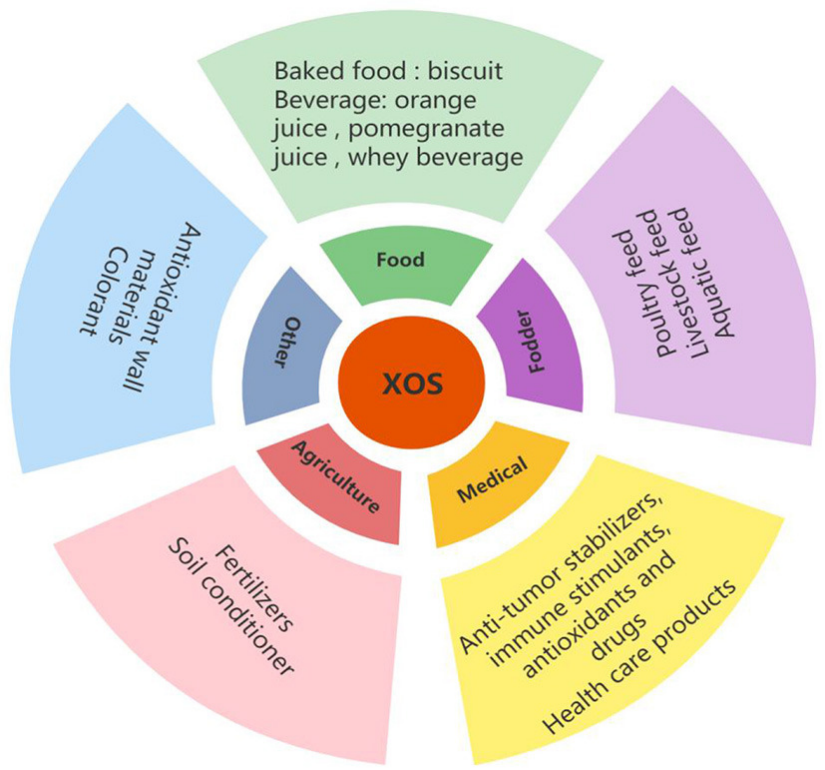
Application of XOS.

Figure 6 showed the different application of XOS. In the food industry, XOS are commonly used as gelling agents, viscosity regulators, foam stabilizers, and tablet adhesives (170). In addition, the use of XOS as fat substitute in dairy products improves the elasticity and hardness of low-fat cheese and improves the storage stability of the cheese (171, 172). Except in the food industry, XOS are also widely used in medicine, agriculture and feed and other field because of their good physical and chemical properties.

### Application of XOS in food

#### Application of XOS in baked food

XOS are widely used in baked foods because of their excellent acid and thermal stability. The moisture retention and water retention of XOS can change the rheological properties of dough and control the best moisture effect to change the taste and appearance of baked food. It was reported that the partial substitution of 5% XOS for sucrose would not change the physical and chemical properties of biscuits but would triple the content of crude fiber and increase the content of dietary fiber by 35%; and the biscuits had the functions of storage stability and prebiotic function (173). The addition of XOS increased the baking characteristics of biscuits, which increased caramel flavor, darker color, and more brittle texture (174). The addition of XOS also increased the sweetness and overall taste intensity of biscuits, indicating that XOS play a role in flavor enhancement in baked products. XOS have proved to be a promising new substitute which can increase the dietary fiber content of cereal biscuits.

#### Application of XOS in beverages

With the addition of XOS to orange juice, pomegranate juice, and whey beverage, the overall sensory acceptance generally improved (170, 175). The strawberry whey beverage with XOS showed an inhibitory effect on the enzymes controlling hypertension and diabetes *in vitro*. With the addition of XOS, the viscosity of the beverage increased, which was attributed to the substantial network formed by hydrogen bonding between XOS and protein and the strong water holding capacity of XOS (176).

### Application of XOS in fodder

XOS could promote the improvement of food-specific characteristics and have been incorporated into animal feed to improve health (20).

In poultry feed, XOS could effectively increase the number of *Lactobacillus* in the ruminant intestine and improve animal digestibility. It was elucidated that adding XOS to grain could improve the feed conversion rate by adjusting the nutrient digestibility and ileal morphology of laying hens, which might be due to the increase of bacterial richness and the change of microbial composition, especially the enrichment of *Lactobacillus* and short-chain fatty acid-producing bacteria and the decrease of *Bacteroides* abundance (177). The addition of XOS to the diet had a positive effect on the growth performance, nutrient digestibility, and SCFA ratio of broilers attacked by coccidia (178). Ribeiro et al. studied the effect of XOS on the performance of broilers and reported that the dietary supplementation of XOS increased the nutritional value of the wheat diet, and the improvement of animal performance was accompanied by the transfer of microbial population settled in the upper gastrointestinal tract (179).

In terms of livestock feed, the addition of 100 g/t XOS in a grain diet could increase the height of jejunal villus, the abundance of *Lactobacillus* and *Bifidobacterium*, as well as the concentration of acetic acid and short-chain fatty acids, and could significantly improve the intestinal ecosystem of Weaned Piglets (180). The addition of XOS had a positive impact on the growth performance, nutrient digestibility, and the proportion of short-chain fatty acids of pigs (181). The supplement of 100 g/t XOS in the growth completion stage of pigs would increase the relative abundance of *Lactobacillus* and short-chain fatty acids and biogenic amines (135). Yin et al. elucidated that the addition of XOS to the diet significantly enhanced the α-diversityof the intestinal microbiota of weaned piglets (157). The addition of XOS increased the villus height: crypt depth ratio in jejunum of weanling piglets. The addition of XOS alone (200 mg/kg) could improve the apparent digestibility of dry matter, nitrogen, and total energy on the 14th day, improve trypsin activity and reduce fecal NH_3_ concentration. On day 14, taking XOS reduced the number of *E. coli* in feces and increased the number of *Lactobacilli* (182).

In terms of aquatic feed, XOS could promote the growth of aquatic animals, reduce the content of serum cholesterol and triglyceride, effectively control blood glucose level and enhance the immunity of aquatic animals. It was reported that after fed Caspian white fish with 3% XOS for 8 weeks, the antibacterial activity and total protein level of skin mucus was significantly improved. After taking XOS into the diet, the total number of intestinal heterotrophic bacteria and lactic acid bacteria increased significantly, which proved the beneficial effect of XOS on different skin mucosal immune parameters (183). Feeding juvenile triploid *O. mykiss* 5.0–10.0 g/kg XOS could increase the number of *Lactobacillus* by promoting intestinal development, limiting intestinal injury and inflammation, and regulating the structure of the intestinal microbial community (182). The high-fat diet supplemented with 1–3% XOS promoted the growth of carp fed with high-fat diet; and XOS could improve the growth, digestive enzymes, antioxidants, and immune response of carp fed with a high-fat diet (184). The appropriate level of XOS supplement could improve the growth performance of grass carp, increase the number of *Lactobacillus* and *Bifidobacterium* and the concentration of short-chain fatty acids, improve the growth performance of grass carp and prevent intestinal cell apoptosis (185). It was reported that added 5% XOS to the diet could significantly improve the growth performance, antioxidant capacity, innate immunity, and hydrophilic bacilli resistance of *Megalobrama amblycephala* (156). Adding XOS to feed European bass (*Dicentrarchusla-brax*) could significantly increase body weight and protein efficiency ratio and feed conversion rate, improve growth, stimulate immunity, and enhance anti-infection ability (186).

### Application of XOS in medical treatment

The unique physiological activities of XOS makes them widely used in medicine for the treatment and prevention of a variety of diseases. In medical care, XOS can be an option in preventing cardiovascular, tumor, and endocrine diseases. XOS can be used as anti-tumor stabilizers, immune stimulants, antioxidants, and drugs. XOS can also be widely added to health care products as functional factors to assist in treating of some human diseases. Through clinical verification, XOS can be combined with traditional Chinese medicine extracts or added to the formula of Western medicine to replace some auxiliary materials such as starch and dextrin, which can strengthen the efficacy of drugs. In addition, XOS can also directly develop health products and enhance the body's physique. According to clinical trials, XOS play an important role in treating diabetes, hypertension, hyperlipidemia, chronic hepatitis, irritable bowel syndrome, chronic gastroenteritis, osteoporosis, pruritus, and otitis (187). Sheu and other studies reported that 8 weeks of XOS as a dietary supplement could effectively improve blood glucose and lipid levels in type 2 diabetes (147). It was found that XOS was beneficial to the reversible change of intestinal microflora in diabetic patients, such as decreasing the growth of *Enterorhabdus, Howardella, SLackia*, and so on (48). At the same time, XOS could also reduce the OGTT-2h (2 h oral glucose tolerance test) of prediabetic patients. It was reported that ID-HWS1000 composed of *Lactobacillus* and *Bifidobacterium*, XOS, and dietary fiber directly improved the discomfort related to defecation, reduced the proportion of vertebrates, increased the proportion of *Bacteroides*, improved the perception of intestinal activity in patients with functional constipation, and produced positive changes (188). XOS supplementation could also improve intestinal function, calcium absorption and lipid metabolism, as well as reduce cardiovascular disease, and colon cancer (23).

### Application of XOS in agriculture

XOS were also known as plant growth regulators (189). XOS could also be used as fertilizers to improve soil activity and promote crop growth. When XOS were used as soil conditioner, the number of soil microorganisms and enzyme activity increased significantly, and the soil ecosystem was improved (190). In XOS treatment, the content of *Brassinolide* (BRS) increased significantly. Some researchers pointed out that BRS could induce the accumulation of zeatin nucleoside in plants to enhance photoprotection by accumulating many cold shock proteins and effectively prevent the accumulation of cold induced proteins (191). Finally, the resistance of plants to low temperature increased (192). Chen et al. reported the effect of XOS on improving salt tolerance of Chinese cabbage (193). XOS had a force to increase root biomass (increased by 69.5%), and the absorption of auxin also increased significantly (194).

### Other applications of XOS

It was reported that adding 3% XOS to a snakehead ball could increase the elasticity of the fishball by 1.32 times without changing the hardness (195). XOS were excellent food additives and could be used as sucrose substitutes in the hydrostatic preparation of high protein meat products. Maillard reaction of soybean protein isolate and XOS could prepare new antioxidant wall materials (196). Soy isolate protein and oligosaccharide conjugate based on Maillard reaction showed excellent potential in microencapsulation of probiotics. Neves and other studies reported that the spray drying blue coloring agent using XOS has shown great potential in many foods as functional ingredients, replacing artificial blue coloring agents and combining the prebiotic characteristics (197). XOS could induce stomatal closure through the production of reactive oxygen species (ROS) and nitric oxide (NO) mediated by salicylic acid signal (198). In addition, in the cosmetics industry, the antioxidant, moisturizing, stabilizer, and emulsifier capabilities of XOS, as well as their ability to restore the microflora, making XOS very attractive (20). Brazil International Flavor Association reported that probiotics and prebiotics were one of the most important active ingredients in the cosmetics market, which could promote the balance of skin microbiota, improve skin resistance, replenish water and alleviate irritation. It was elucidated that XOS could avoid protein denaturation during frozen storage; shrimp soaked in XOS solution (3.0% w/v) had better water retention, stability of myofibrillar protein, and excellent texture characteristics (199). The mechanism of protein stability was described by the hydrogen bond between XOS and the polar residues of muscle protein and by limiting the fluidity of water to avoid the growth of ice crystals.

## Conclusion

XOS are highly effective prebiotic nutritional oligosaccharudes, which can be produced by hydrolysis of hemicellulose, a rich component in agricultural residues rich in xylan. At present, the preparation of XOS mainly includes acid hydrolysis, autohydrolysis, and chemical enzyme synthesis. Although XOS can be produced by chemical hydrolysis; enzymatic hydrolysis has significant advantages because it usually does not produce byproducts, which is very important for the application of XOS. In the industrial environment, the need for biomass pre-treatment and the relatively low efficiency of subsequent enzymatic hydrolysis limit the yield of XOS. Therefore, the successful production of XOS requires strict and optimized conditions.

The existing methods optimize the preparation of XOS to a certain extent and improve the preparation efficiency. In recent years, the industrial application of xylan, a byproducts of agricultural products, has been greatly limited due to its high content of impurities, and the product quality is not easy to control. Therefore, the refining, separation, and purification of XOS have also become the key to their subsequent industrial application. At present, the development of XOS has not reached its peak, and as a new generation of functional sugars, XOS have not been fully used. These production optimizations have promoted the application and development of XOS and laid the primary theoretical foundation for large-scale popularization and use in the future.

The existing physiological activity studies of XOS are carried out in animals and can only speculate on the effect on human body according to the obtained data. The actual effects need further experimental verification. In the preparation process of XOS, the effects of different raw materials on the structure and physiological activity of XOS cannot be determined; further research is needed.

XOS have an auspicious future. With the continuous in-depth development and promotion of the health care industry, there are more and more customers' needs. The advantages of XOS are less addition, good stability, and high selectivity, which is in line with the general demand that capsules, tablets, and other dosage forms are easier to carry and take. These advantages are unmatched by other oligosaccharides. In addition, as relatively new feed additive, they also have excellent performance in bacteriostasis. XOS can further maintain the health and productivity of animals. With many countries have enacted laws, and more and more antibiotics are avoided outside the scope of feed additives, including the continuous improvement of various policies and the gradual diversification and innovation of industrial development, there will be a new opportunity for the sustainable development of the XOS industry.

## Author contributions

FY: conceptualization, funding acquisition, and writing—original draft. ST: formal analysis, investigation, resources, and writing—review and editing. KD: investigation and resources. XX: investigation, methodology, and supervision. PG: investigation and methodology. ZC: investigation and supervision. All authors contributed to the article and approved the submitted version.

## Funding

This research was supported by the Cultivation Programme for Young Backbone Teachers in Henan Province (2021GGJS059) and funded by Natural Science Innovation Fund Support Program from Henan University of Technology (2021ZKCJ12).

## Conflict of interest

The authors declare that the research was conducted in the absence of any commercial or financial relationships that could be construed as a potential conflict of interest.

## Publisher's note

All claims expressed in this article are solely those of the authors and do not necessarily represent those of their affiliated organizations, or those of the publisher, the editors and the reviewers. Any product that may be evaluated in this article, or claim that may be made by its manufacturer, is not guaranteed or endorsed by the publisher.
